# Combined evaluation of blood flow and tissue perfusion in diabetic feet by intra-arterial dynamic 4DCT imaging

**DOI:** 10.1186/s41747-023-00352-x

**Published:** 2023-07-26

**Authors:** Pieter T. Boonen, Nico Buls, Jef Vandemeulebroucke, Gert Van Gompel, Frans Van Den Bergh, Tim Leiner, Dimitri Aerden, Johan de Mey

**Affiliations:** 1 Department of Radiology, Vrije Universiteit Brussel (VUB), Laarbeeklaan 101, 1090 Brussels, Belgium; 2 Department of Electronics and Informatics (ETRO), Vrije Universiteit Brussel (VUB), Pleinlaan 2, 1050 Brussels, Belgium; 3 Kapeldreef 75, 3001 Leuven, Belgium; 4 Department of Radiology, Mayo Clinic, 200 1st St SW, Rochester, MN 55901 USA; 5 Department of Vascular Surgery, Vrije Universiteit Brussel (VUB), Laarbeeklaan 101, 1090 Brussels, Belgium

**Keywords:** Angiography (digital subtraction), Diabetic foot, Ischemia, Perfusion imaging, Tomography (x-ray computed)

## Abstract

Critical limb ischemia is associated with high mortality and major amputations. Intra-arterial digital subtraction angiography (IADSA) has been the reference standard but has some shortcomings including the two-dimensional projection and the lack of tissue perfusion information. The aim of this exploratory study is to examine four-dimensional computed tomography (4DCT) angiography and perfusion imaging using low-volume intra-arterial contrast injections for an improved anatomic and hemodynamic assessment in patients with foot ulcers. Three patients underwent a low-volume (2 mL) intra-arterial contrast-enhanced 4DCT examination combined with a diagnostic IADSA. An automated assessment of blood flow and tissue perfusion from the 4DCT data was performed. Vascular structures and corresponding blood flows were successfully assessed and correlated well with the IADSA results. Perfusion values of the affected tissue were significantly higher compared to the unaffected tissue. The proposed 4DCT protocol combined with the minimal usage of contrast agent (2 mL) provides superior images compared to IADSA as three phases (arterial, perfusion, and venous) are captured. The obtained parameters could allow for an improved diagnosis of critical limb ischemia as both the proximal vasculature and the extent of the perfusion deficit in the microvasculature can be assessed.

**Relevance statement**

Intra-arterial 4DCT allows for assessing three phases (arterial, perfusion and venous) using minimal contrast (2 mL). This method could lead to an improved diagnosis of critical limb ischemia as both proximal vasculature and the extent of the perfusion deficit are assessed.

**Trial registration**

ISRCTN, ISRCTN95737449. Registered 14 March 2023—retrospectively registered, https://www.isrctn.com/ISRCTN95737449

**Key points**

• Three phases (arterial, perfusion, and venous) are obtained from 2 mL intra-arterial 4DCT.

• The obtained hemodynamic parameters correlated well with the IADSA findings.

• 4DCT surpassed IADSA in terms of assessment of venous blood flow and inflammatory hyperperfusion.

• The assessment of tissue perfusion could lead to optimizing the revascularization strategy.

**Graphical Abstract:**

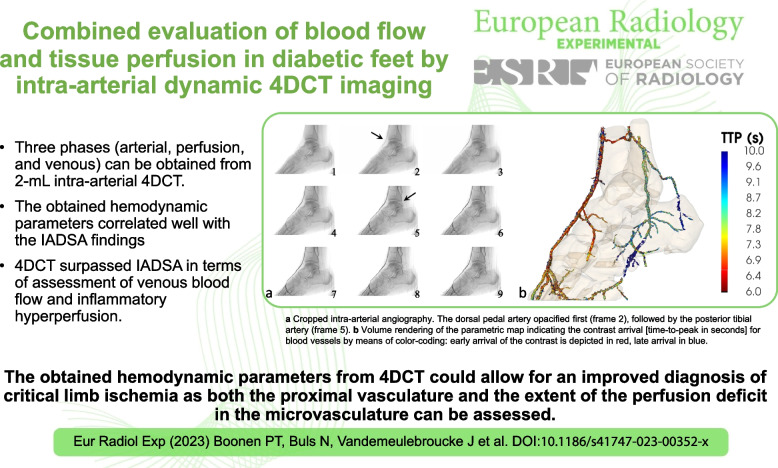

**Supplementary Information:**

The online version contains supplementary material available at 10.1186/s41747-023-00352-x.

## Background

Peripheral arterial disease (PAD) is a chronic atherosclerotic process which causes partial or complete obstruction of peripheral arteries, predominantly in the lower extremities [1, 2]. It is the third leading cause of atherosclerotic morbidity [3] and patients with diabetes have a two- to four-fold increased risk of developing PAD compared to nondiabetic individuals [4]. It is estimated that 20% of PAD patients will eventually develop critical limb ischemia, which is associated with high mortality and major amputation in people with diabetes [3, 5].

Atherosclerotic disease in diabetic foot patients is characterized by a predilection of occlusive disease in below-the-knee arteries and pronounced media calcinosis. Many characteristics are considered when devising an optimal revascularization strategy: arterial anatomy, atherosclerotic lesion morphology, localization, extent, and foot tissue perfusion [6]. Intra-arterial digital subtraction angiography (IADSA) has been the gold standard for examining small and highly calcified arteries, providing morphological and hemodynamic information [7]. Although wound healing is impacted by perfusion deficits and assessing it could be used to evaluate foot ulcers and determine whether revascularization is required, IADSA does not provide information on tissue perfusion. Additionally, lesions are only evaluated in 2D projections, leading to potential underestimations of occlusive severity due to superposition errors [7, 8].

The examination of PAD in the below-the-knee arteries using conventional computed tomography (CT) is restricted due to the artifacts caused by the calcifications combined with the lower resolution of CT compared to DSA [9]. In addition, people with diabetes and renal impairment are at a higher risk of developing contrast medium-induced nephropathy after being administered iodinated contrast media intravenously [10].

In recent years, there has been an increased interest in dynamic four-dimensional computed tomography angiography (4DCT) imaging. The advent of wide beam CT scanners allows to perform multiple CTA acquisitions covering up to 160 mm of anatomy at high temporal resolution. Several feasibility studies for 4DCTA applications have been reported by Rajiah et al. [11], including evaluation of the lower legs [12], blood flow perfusion assessment in the feet [13], and evaluation of endoleaks [14]. However, these studies exclusively involved intravenous contrast injections, typically ranging from 40 to 80 mL [11]. Local intra-arterial contrast injections, however, could allow for a vascular assessment using lower contrast volumes.

This paper examines a new methodology for assessing critical limb ischemia by employing dynamic 4DCT angiography and perfusion imaging (CTP) in conjunction with IADSA, using a minimal volume of intra-arterial iodinated contrast. To this end, we describe the combined evaluation of blood flow and tissue perfusion in three diabetic foot patients with critical limb ischemia using intra-arterial dynamic 4DCT.

## Methods

### Interventional imaging protocol

Between January 2021 and August 2021, three participants with diabetic foot and a high suspicion of critical limb ischemia received a dynamic 4DCT examination combined with a diagnostic IADSA examination as part of their vascular workup at the UZ Brussel diabetic foot clinic. Both the IADSA and dynamic 4DCT acquisitions of ankle and feet were performed on a 4DCT system (Alphenix 4DCT, Canon medical systems, Otawara, Tochigi, Japan). This hybrid system combines an interventional C-arm system with a wide beam CT scanner on rails. The participants were in supine position and a Simmons-2, five French Catheter (Cook Medical, Indiana, USA) was introduced through femoral access after skin preparation under local anesthesia. Ten milliliters of iodinated contrast (370 mg I/mL, Ultravist, Bayer Healthcare, Ota Tokyo, Japan) diluted with 5 mL saline was injected proximal to the aorta bifurcation at a rate of 4.0 mL/s. IADSA acquisitions were performed at a rate of 3 frames/s.

Immediately after IADSA, the CT gantry was positioned over the ankle and feet to perform a combined CT and CTP imaging protocol consisting of multiple 160-mm axial series. The tip of the catheter was positioned in the distal abdominal aorta. First, a 40-s continuous acquisition was performed, followed by 16 repeated acquisitions with 2-s interphase delay and seven acquisitions at 5-s interval. Finally, 6 additional acquisitions were made at 15-s interval to evaluate tissue perfusion. Reconstructing the continuous acquisitions at a 0.1-s temporal resolution rendered 431 three-dimensional (3D) CT volumes with 0.5 mm × 0.5 mm × 0.5 mm spacing over the total scan duration of 194 s.

Scan parameters were: tube voltage 100 kVp, tube current 52 mA and rotation time 0.35 s. Total CT dose index volume was 216.07 mGy. For the combined CTA and CTP series, 2 mL of contrast was diluted with 18 mL saline, administered intra-arterially at 4.0 mL/s during 5 s. CT acquisitions started simultaneously with contrast administration. The effective dose of the IADSA examination and the dynamic 4DCT series were 0.02 mSv and 0.91 mSv, respectively (NCIDose, ICRP-103 [15]).

### Participant description

The following participant description is based on the IADSA results, assessed by a vascular surgeon (D.A.) with 16 years of experience.

**Fig. 1:**
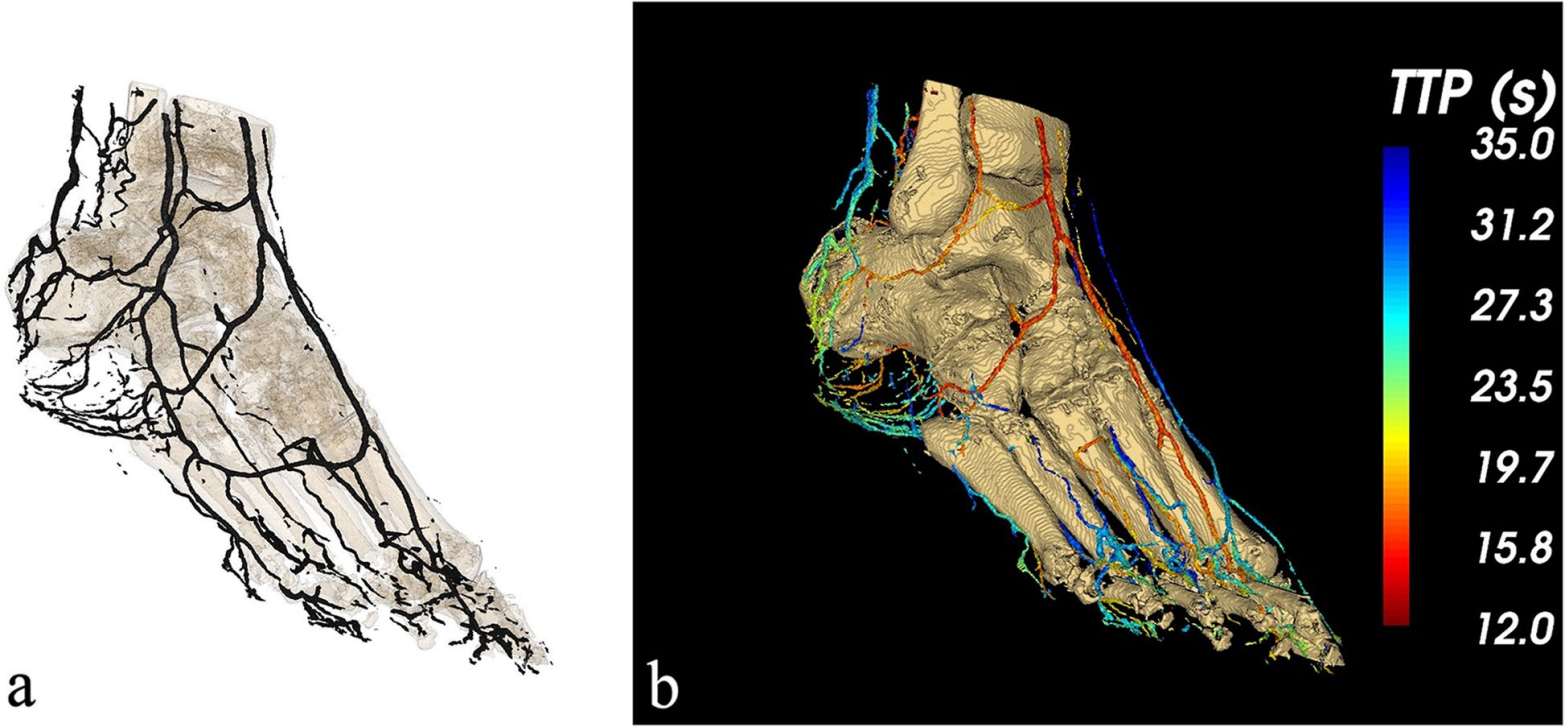
**a** Rendering of the enhanced arterial segments derived from the 4DCT data (participant number 1) at 18. 9 s after contrast injection. **b** Volume rendering of the parametric map indicating the contrast arrival (time-to-peak in seconds) for blood vessels by means of color-coding: early arrival of the contrast is depicted in red, late arrival in blue. *4DCT* Four-dimensional computed tomography

Participant number 1 (male, 88 years old) presented with a noninfected, superficial ulcer at the lateral side of the right heel. All three below-the-knee arteries appeared occluded and numerous collaterals were present. Due to incompressible arteries, the ankle-brachial index could not be obtained. Late scanning revealed fair enhancement of the peroneal artery, which connected to the plantar posterior tibial artery via a side branch (Fig. 1).

**Fig. 2:**
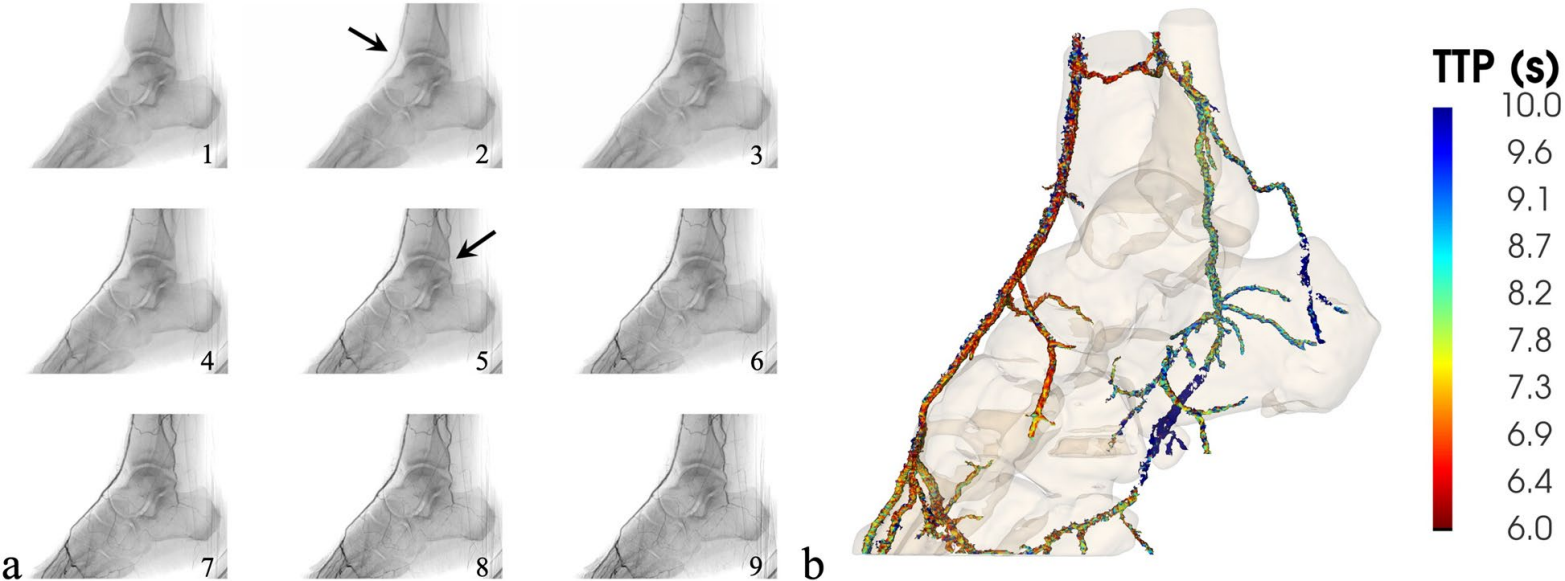
**a** Cropped intra-arterial angiography of participant number 2. The dorsal pedal artery opacified first (frame 2), followed by the posterior tibial artery (frame 5). **b** Volume rendering of the parametric map indicating the contrast arrival [time-to-peak in seconds] for blood vessels by means of color-coding: early arrival of the contrast is depicted in red, late arrival in blue

For the participant number 2 (male, 58 years old), wound characteristics were typical for occlusive disease (dry, sharply delineated necrosis at the toes) and the ankle-brachial index could not be obtained due to incompressible arteries caused by vessel wall calcifications. Multiple high-grade stenoses were present in the anterior tibial artery, but the artery remained patent over its entire length. The posterior tibial artery however showed a short occlusion at its origin, becoming merely stenotic more distally (Fig. 2).

**Fig. 3:**
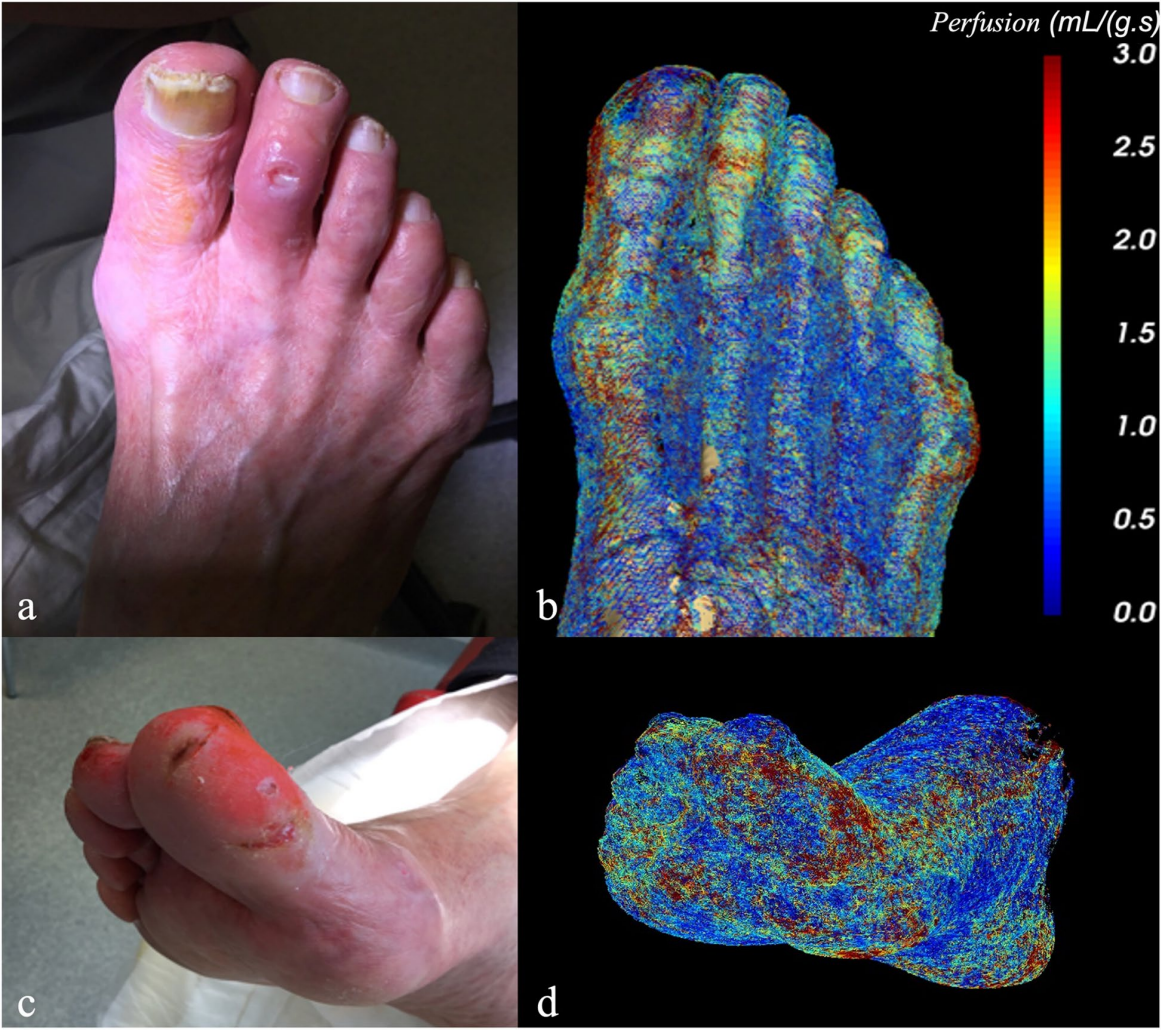
**a**, **c** Participant number 3 presenting several necrotic ulcers at the first and second toe. **b**, **d** Volume rendering of the parametric blood flow map indicating the perfusion values (mL/g/s) derived from 4DCT by means of color-coding: low perfusion values are depicted in blue, high values in red. *4DCT* Four-dimensional computed tomography

Participant number 3 (female, 84 years old) demonstrated several small necrotic ulcers at the first and second right toe. No inflammatory signs were present, and the ankle-brachial index could not be obtained due to vessel wall calcification. Occlusive disease manifested exclusively in below-the-knee arteries: both tibial arteries were highly stenotic proximally, then became occlusive 10 cm further downstream. The peroneal artery remained patent up until the ankle. No foot arteries could be discerned during the IADSA, the tibial arteries filled in a very late stage but appeared highly stenotic and gracile (Fig. 3).

### Image processing

Image processing of the 4DCT angiography and CTP data was carried out using in-house developed software written in Python and C +  + , using the Insight Segmentation and Registration Toolkit [16] and the Elastix library [17].

To correct for patient motion, all images were registered to the baseline image (*t* = 0 s) by pairwise rigid registration. Firstly, the bones were automatically extracted from the first baseline image (*t* = 0 s) using Otsu thresholding, mathematical morphology, and connected component analysis. Secondly, soft tissue was segmented using a minimum threshold of 10 HU. From these segmentations, five volumes of interest (VOIs) were automatically generated in the dorsal, plantar (lateral and medial) and calcaneal (lateral and medial) aspects of the foot according to the “angiosome” concept [18]. This principle divides the foot into five regions fed by source arteries. To segment the blood vessels, the local variance over time was computed in each voxel. As the propagating contrast bolus induces a strong fluctuation in intensity over time, the variance becomes maximal over the blood vessels, while yielding low values for the muscles and bones. Both arteries and veins were then automatically segmented Otsu thresholding, mathematical morphology, and connected component analysis on the variance image. Finally, five source arteries were identified and five VOIs without calcifications were segmented from, respectively, the dorsal pedal artery, lateral plantar artery, medial plantar artery, lateral calcaneal artery, and medial calcaneal artery.

### Quantitative image analysis

Arterial anatomy and morphological characteristics of all atherosclerotic lesions were assessed. Specific attention was given to the hemodynamic properties of the contrast bolus traveling through the ankle- and foot arteries. Time attenuation curves were generated using cubic polynomials to determine the time-to-peak (TTP) values corresponding to the time of maximal enhancement in the blood vessels and VOIs. Applying these TTP values, 3D parametric maps were rendered to visualize the blood flow along the centerline of the corresponding arteries. In addition, a voxel-wise assessment of the blood flow through the microvasculature was performed using the maximum slope method to compute tissue perfusion (mL/g/s) [19]. Voxel-by-voxel parametric maps were created from the TTP values and the perfusion data using 3Dslicer [20]. In addition, VOIs of the wounds and adjacent healthy tissue were manually segmented and tissue perfusion values (mL/g/s) were compared using a Mann–Whitney *U* test (SPSS, IBM V27, New York, USA). Differences with *p* values < 0.05 were considered statistically significant.

## Results

### Blood vessel anatomy and hemodynamic characteristics

Dynamic 4DCT series allowed for selecting the phase of maximal enhancement in the arterial segments. A 3D volume rendering of the arterial enhancement phase of participant number 1, acquired at 18.9 s after contrast injection, is shown in Fig. 1a. Such pseudoangiography, of which a single two-dimensional view closely resembles a DSA image, provides a straightforward volumetric visualization of the arterial segment. Representation of multiple temporal phases in a single image is shown by the parametric TTP map in Fig. 1b. The extended acquisition time also allowed the evaluation of blood flow in the veins. Consequently, the TPP map can be used to distinguish between arteries and veins, the first being displayed in red and yellow (12–19 s), the latter in green and blue (23–25 s).

Figure 2a shows the sagittal plane of the intra-arterial angiogram obtained from the second participant. From the IADSA, it was apparent that the contrast arrived firstly in the dorsal pedal artery, which subsequently provided flow towards the plantar arch, which filled retrogradely. Compared to the dorsal pedal artery, the posterior tibial artery filled seconds later in an antegrade way. The peroneal artery, which was affected most by occlusive disease, was of very small caliber and only filled after an extended period.

Analysis of the 4DCT data showed high correspondence with the IADSA, both on morphological and hemodynamical level (Fig. 2b). Morphologically, the characteristics of the segmented arteries are very detailed and identical to the angiogram. Hemodynamically, lower TTP values for the dorsal pedal artery (TTP = 7.7 s) are apparent compared to the posterior tibial artery (TTP = 9.2 s) and the peroneal artery (TTP = 10.2 s). These differences in TTP correspond to the differences observed from the IADSA images. In addition, the retrograde filling of the plantar arch is more prominent compared to the IADSA images.

### Soft tissue perfusion

Dynamic 4DCT series (Supplementary multimedia file) allowed for tracking the contrast bolus through blood vessels and soft tissue. Good agreement between the necrotic foot ulcers for participant number 3 and the derived perfusion values of the dermis can be seen in Fig. 3. The areas with elevated perfusion values, indicated in red, are more dispersed, but the highest concentrations are located at the first toe, second toe, fifth toe, and the medial calcaneal. This corresponds well with the locations of the different foot ulcers. The results of the perfusion values for affected tissue (soft tissue that harbors the ulcer or necrotic lesion) and surrounding unaffected tissue are shown in Table 1. For all three participants, the perfusion values of the affected tissue were significantly higher compared to the unaffected tissue.

**Table 1:** Median blood flow perfusion values (mL/g.s) for wounds and adjacent unaffected tissue derived from 4DCT imaging using the arterial input function and slope method

	Unaffected angiosome		Affected angiosome		
Participants	Perfusion (mL/g.s)	95% CI	Perfusion (mL/g.s)	95% CI	*p* value
Participant 1	0.87	[0.83–0.92]	1.44	[1.32–1.54]	< 0.001
Participant 2	1.28	[1.20–1.36]	3.89	[3.68–4.10]	< 0.001
Participant 3	0.96	[0.88–1.04]	4.81	[4.79–5.05]	< 0.001

*p* values were calculated by applying a Mann–Whitney *U* test. *CI* Confidence interval

## Discussion

This exploratory study investigated a new technique for assessing critical limb ischemia and tissue perfusion. The proposed methodology combines an IADSA examination with dynamic 4DCT to allow a more extensive hemodynamical assessment of critical limb ischemia as three phases (arterial, perfusion, and venous) are captured in 3D with minimal usage of contrast (2 mL) and with acceptable radiation dose (0.91 mSv). To the best of our knowledge, this is the first study that reports a combined acquisition of CTA and CTP imaging data of the feet by intra-arterial injection using limited contrast volume (2 mL) together with an IADSA procedure of the lower extremities.

The 4DCT data correlated well with IADSA findings in several regards. Firstly, the arterial anatomy and morphology of the occlusive lesions could easily be discerned on the CT images. Secondly, arterial dominancy (arterial segment in the foot that receives the blood bolus first) corresponded well with IADSA findings. Thirdly, the flow direction (retrograde filling by collaterals) could likewise be observed on 4DCT. It surpasses IADSA in two regards: visualization of venous flow and inflammatory hyperperfusion. While this can be observed using IADSA (by late filling of venous vessels and blush in highly inflamed tissue), the 4DCT seemed more sensitive in these three participants.

The 194-s acquisition time, combined with the high temporal resolution, allowed evaluation of both arterial and venous blood flow. Parametric maps of the TTP can be generated and the delay in contrast arrival time caused by proximal stenoses can be observed. In addition, CTP data represents the blood flow in the microvasculature. In all three participants, foot ulcers yielded significantly higher perfusion values compared to adjacent unaffected tissue. This information could be valuable in the decision of revascularization strategy and could also be used as a predictor for wound healing and its evaluation pre-and post-intervention.

Previous studies [13, 21] have demonstrated the feasibility of pedal blood flow measurements using 4DCT. Our results are consistent with their findings. However, in these studies, the contrast agent was administered intravenously. The authors reported the usage of contrast agent (47 mL and 100 mL) as a major limitation as the presence of PAD is one of the risk factors associated with contrast material-induced nephrotoxicity. Acquiring the 4DCT images in conjunction with an IADSA examination with intra-arterial contrast administration drastically reduces the amount of contrast agent needed. Furthermore, the arterial and venous phases were not quantified nor visualized in these studies while being crucial for accurately assessing tissue perfusion. The reported radiation dose for these studies was 0.31 mSv (effective dose) and 12.53 mSv (equivalent dose), respectively.

The following limitations of our technical development study merit consideration. Firstly, the 4DCT acquisitions were not repeated due to the radiation exposure. Therefore, the variation in successive measurements could not be assessed. Secondly, the proposed 4DCT acquisition protocol cannot be used in clinical studies as it should be optimized in terms of the radiation dose. Evaluating the effect of increasing the interphase delay on the assessed parameters could allow for a decreased radiation dose while maintaining image quality. This is outside the scope of this technical development study. Thirdly, the 3D parametric maps were not obtained in real time as the method includes manual identification of the source arteries and a computation time of approximately two hours due to the image registration step. Finally, we applied a case-study approach to this exploratory study, limiting the sample size to three participants.

In conclusion, we proposed a new methodology for assessing critical limb ischemia by employing intra-arterial 4D CTA and CTP imaging, using a minimal volume of contrast (2 mL). We established an automated workflow to assess the anatomy of the blood vessels, the hemodynamics of the blood flow (arterial and venous), and the tissue perfusion. The obtained hemodynamic parameters could allow for an improved diagnosis of critical limb ischemia as both the proximal vasculature and the extent of the perfusion deficit in the microvasculature can be assessed.

## Supplementary Information

**Additional file 1:** **Supplemental movie 1.** 4D rendering of the passing contrast bolus throughthe blood vessels and soft tissue, derived from the 4DCT data.

## Data Availability

Data generated or analyzed during the study are available from the corresponding author by request.

## Abbreviations

3D: Three-dimensional
4DCT: Four-dimensional CT
CT: Computed tomography
CTA: CT angiography
CTP: CT perfusion
IADSA: Intra-arterial digital subtraction angiography
PAD: Peripheral arterial disease
TTP: Time to peak
VOI: Volume of interest
